# Supplementary material for: A hepatic network of dendritic cells mediates CD4 T cell help outside lymphoid organs
Source: Nat Commun. 2024 Feb 10;15:1261. doi: 10.1038/s41467-024-45612-5 (PMC10858872; doi:10.1038/s41467-024-45612-5)
Supplement: Supplementary file 1 — Supplementary Information [file 41467_2024_45612_MOESM1_ESM.pdf]

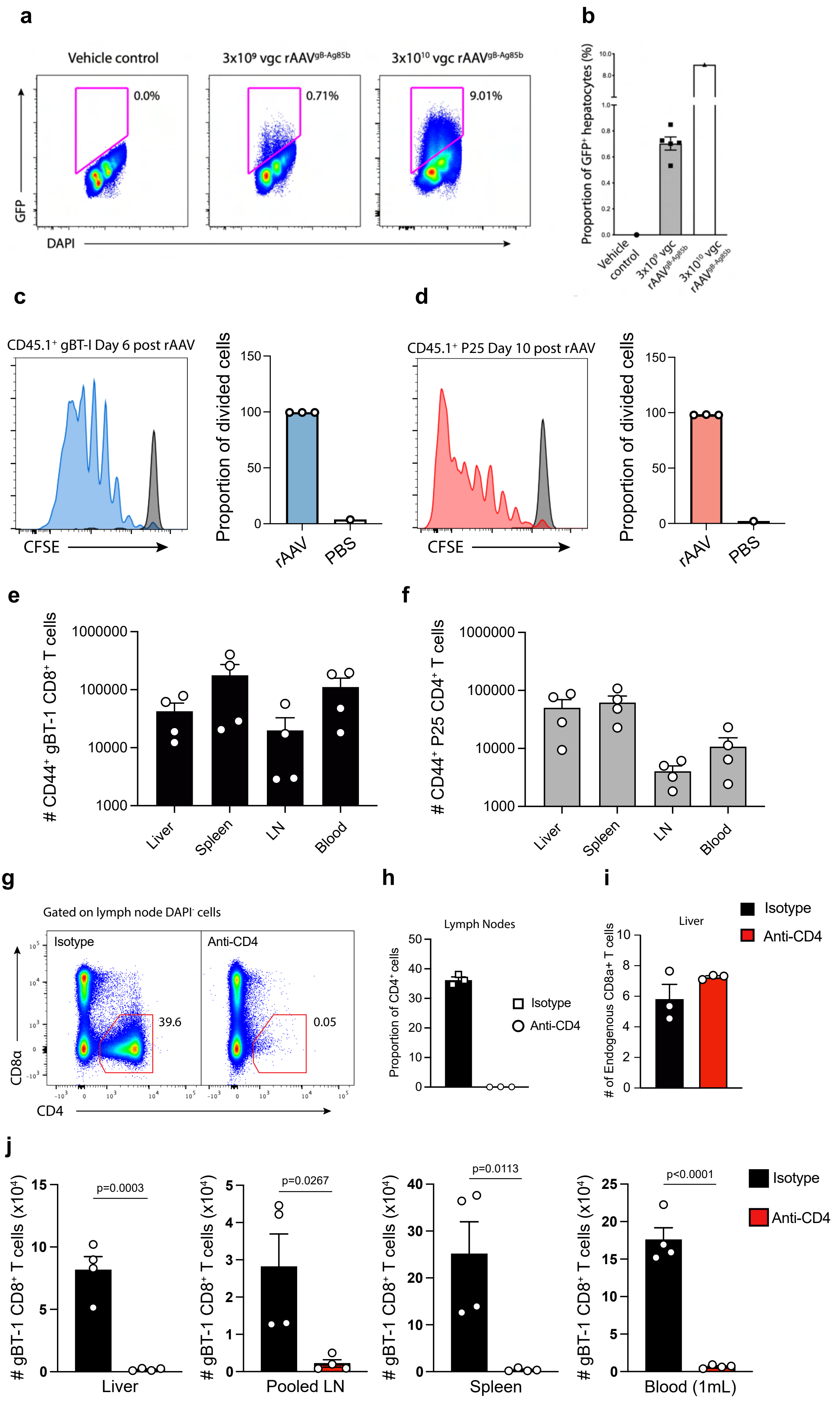
a
Vehicle control
3x10⁹ vgc rAAV gB-Ag85b
3x10¹⁰ vgc rAAV gB-Ag85b
0.0%
0.71%
9.01%
GFP
DAPI
b
Proportion of GFP+ hepatocytes (%)
Vehicle control
3x10⁹ vgc rAAV gB-Ag85b
3x10¹⁰ vgc rAAV gB-Ag85b
c
CD45.1+ gBT-I Day 6 post rAAV
CFSE
Proportion of divided cells
rAAV
PBS
d
CD45.1+ P25 Day 10 post rAAV
CFSE
Proportion of divided cells
rAAV
PBS
e
CD44+ gBT-1 CD8+ T cells
Liver
Spleen
LN
Blood
f
CD44+ P25 CD4+ T cells
Liver
Spleen
LN
Blood
g
Gated on lymph node DAPI- cells
Isotype
Anti-CD4
39.6
0.05
CD8α
CD4
h
Lymph Nodes
Proportion of CD4+ cells
Isotype
Anti-CD4
i
Liver
# of Endogenous CD8a+ T cells
Isotype
Anti-CD4
j
# gBT-1 CD8+ T cells (x10⁴)
p=0.0003
Liver
p=0.0267
Pooled LN
p=0.0113
Spleen
p<0.0001
Blood (1mL)
Isotype
Anti-CD4

Supplementary Figure 1. A robust $CD4^+$ T cell dependent gBT-1 $CD8^+$ T cell response is generated following *in vivo* $rAAV^{gB-Ag85b}$ treatment. Related to Figure 1.

(**a**) Flow cytometric plots of primary hepatocytes isolated from the livers of C57BL/6 mice injected i.v. with PBS, $3x10^9$ or $3x10^{10}$ $rAAV^{gB-Ag85b}$ viral genome copies (vgc). Hepatocytes were stained with DAPI and the proportion of transduced hepatocytes was analyzed by assessing GFP expression. (**b**) Proportion of GFP^+ transduced hepatocytes in C57BL/6 mice treated with PBS or$AAV^{gB-Ag85b}$ as described in (b). (**c-d**) Donor transgenic $CD45.1^+CD8^+$ gBT-1 (c) and $CD45.1^+CD4^+$ P25 T cells (d) recognized cognate antigen expressed in rAAV-transduced hepatocytes. C57BL/6 mice received adoptively transfer of CFSE labelled gBT-1 $CD8^+$ T cells or CFSE labelled P25 $CD4^+$ T cells and, one day later, were treated with $3x10^9$ vgc $rAAV^{gB-Ag85b}$ or PBS i.v. At 6- or 10-days, leukocytes were isolated from the liver draining LNs of recipient mice and proliferation was assessed via CFSE dilution. Left panels: Representative flow cytometric histogram depicting CFSE dilution in proliferating Tg $CD8^+$ T cells (c) and Tg $CD4^+$ T cells (d); Right panels: Quantification of the proportion of cells that have undergone at least 1 division (right panel). (**e-f**) Absolute number of memory gBT-1 $CD8^+$ (e) and memory P25 $CD4^+$ T cells (f) 8 weeks post rAAV treatment, in the livers, spleens, pooled LNs (LN) and blood (1mL) of recipient C57BL/6 mice that had received adoptive transfer of 10,000 gBT-1 and 10,000 P25 T cells and had been treated with $3x10^9$ vgcs $rAAV^{gB-Ag85b}$ one day later. (**g-h**) Proportion of $CD4^+$ T cells in the lymph nodes of mice treated with GK1.5 or isotype control. Representative FACS plots (g) and quantification (h). (**i**) The total number of $CD8\alpha^+$ T cells in the liver of C57BL/6 mice treated with GK1.5 or isotype control. (**j**) Absolute number of memory gBT-1 $CD8^+$ T cells isolated from the liver, pooled lymph nodes, spleen and blood (1mL) of isotype control and anti-CD4 (GK1.5) treated recipient mice 56 days post rAAV treatment. Data are representative of two independent experiments with n=5 mice per 3e9 rAAV treated group and n=1 mouse per control group (a-b), n=3 (c, d, g, h, i), n=4 mice per group (e, f, j). Error bars indicate ± SEM. Analyzed with a two tailed unpaired Student's *t* test. Source data are provided as a Source Data file.

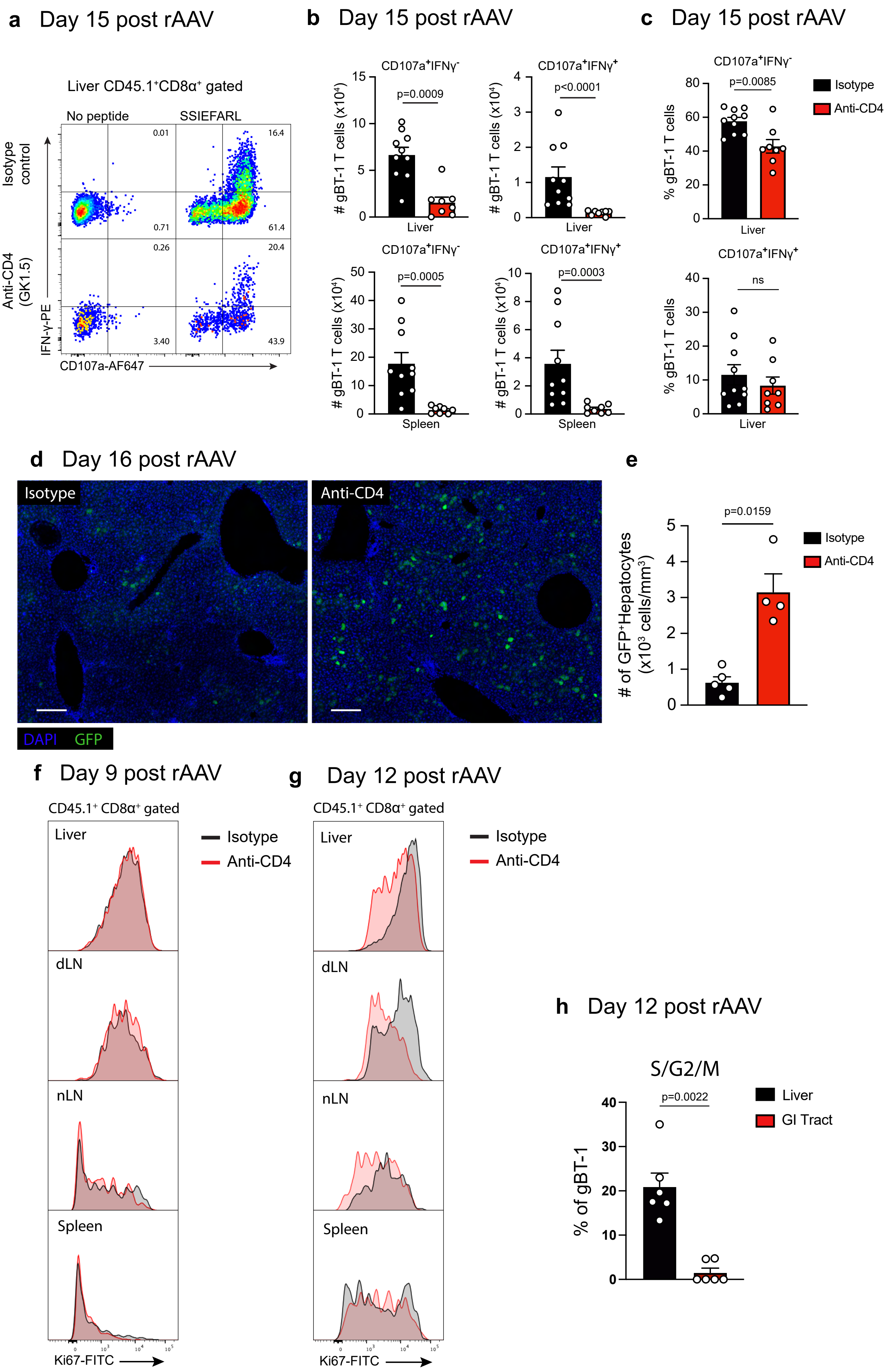
a
Day 15 post rAAV
Liver CD45.1⁺CD8α⁺ gated
No peptide
SSIEFARL
Isotype control
Anti-CD4 (GK1.5)
0.01
16.4
0.71
61.4
0.26
20.4
3.40
43.9
IFN-γ-PE
CD107a-AF647
b
Day 15 post rAAV
CD107a⁺IFNγ⁻
CD107a⁺IFNγ⁺
p=0.0009
p<0.0001
p=0.0005
p=0.0003
# gBT-1 T cells (x10⁴)
Liver
Spleen
c
Day 15 post rAAV
CD107a⁺IFNγ⁻
CD107a⁺IFNγ⁺
p=0.0085
ns
% gBT-1 T cells
Liver
Isotype
Anti-CD4
d
Day 16 post rAAV
Isotype
Anti-CD4
DAPI
GFP
e
p=0.0159
# of GFP⁺Hepatocytes (x10³ cells/mm³)
Isotype
Anti-CD4
f
Day 9 post rAAV
CD45.1⁺ CD8α⁺ gated
Liver
dLN
nLN
Spleen
Ki67-FITC
Isotype
Anti-CD4
g
Day 12 post rAAV
CD45.1⁺ CD8α⁺ gated
Liver
dLN
nLN
Spleen
Ki67-FITC
Isotype
Anti-CD4
h
Day 12 post rAAV
S/G2/M
p=0.0022
% of gBT-1
Liver
GI Tract

Supplementary Figure 2. CD4 help influences the density and distribution of effector gBT-1 T cells in the liver 15-days post rAAV treatment. Related to Figure 2.

(**a**) Representative FACS plot demonstrating ability of intrahepatic effector $CD8^+$ gBT-1 T cells to degranulate and produce IFN-γ after ex vivo peptide stimulation. (**b-c**) Comparison of functional effector gBT-1 T cell numbers isolated from the liver and spleen at 15-days post rAAV treatment of CD4-depleted (red) versus isotype control (black) treated animals. (**d-e**) Representative IF images (d) and quantification (e) of the number of GFP^+ hepatocytes in the livers of recipient mice treated with isotype control (left image; black) or GK1.5 (right image; red) 16-days post rAAV. (**f-g**) Representative histograms showing the expression level of Ki67 in gBT-1 T cells isolated from CD4 depleted and isotype control treated recipient mice 9-days (f) and 12-days (g) post $rAAV^{gB\text{-}Ag85b}$ treatment. (**h**) Quantification of the proportion of gBT-1 T cells in S/G2/M phases of the cell cycle in the liver and GI tract 12-days post rAAV. Data are representative of two independent experiments. n=8 or 10 (b, c), n=4 or 5 (d-g), or n=6 (h) mice per group. Error bars indicate ± SEM. Analyzed with a two-tailed unpaired Mann Whitney U test (b, c, e, h). Scale bars 200μm (d). Source data are provided as a Source Data file.

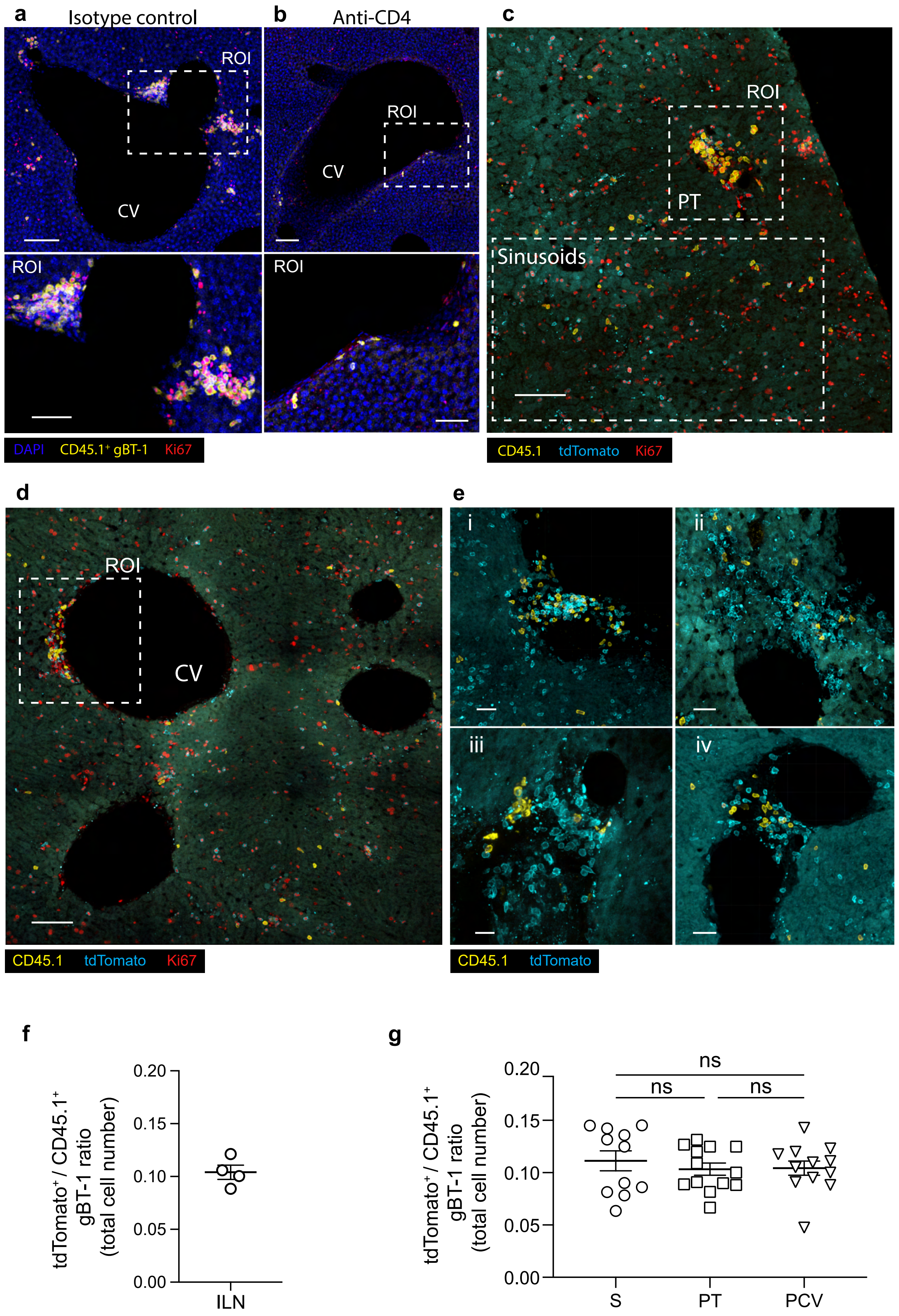

a
Isotype control
b
Anti-CD4
c
ROI
CV
ROI
DAPI
CD45.1+ gBT-1
Ki67
PT
Sinusoids
CD45.1
tdTomato
Ki67
d
e
i
ii
iii
iv
f
tdTomato+ / CD45.1+ gBT-1 ratio (total cell number)
0.20
0.15
0.10
0.05
0.00
ILN
g
ns
S
PT
PCV

Supplementary Figure 3. CD4 help promotes peripheral expansion of CD8^{+} T cells in portal tracts and PCV regions of the liver. Related to Figure 3.

(**a-b**) Representative confocal images of Ki67^{+} gBT-1 T cell clusters in PCV regions of the liver 12 days post rAAV treatment of recipient animals administered isotype control (a) or anti-CD4 (b) mAbs showing that intrahepatic gBT-1 T cells responding in the presence of CD4 T cells were actively proliferating in PCV regions of the liver. (**c-d**) Representative confocal images of Ki67^{+} gBT-1 T cell clusters within portal tracts (c) and PCV regions (d) that contain a ratio of tdTomato^{+}: CD45.1^{+} cells inconsistent with the original 1:10 CD45.1/tdTomato ratio inoculated, indicating local proliferation. A high magnification view of the ROI PT (portal tracts) and sinusoids is shown in Figure 3. (**e**) Additional representative confocal images of gBT-1 T cell clusters within portal tracts from n=4 mice containing varying ratios of tdTomato^{+}: CD45.1^{+} cells. Recipient C57BL/6 mice received an initial adoptive transfer ratio of 9,000 tdTomato^{+} (cyan) and 1,000 CD45.1^{+} (yellow) gBT-1 T cells. Scale bar 40µm (i), 40µm (ii), 20µm (iii) and 40µm (iv). (**f**) Quantification of the ratio of total tdTomato^{+}/CD45.1^{+} gBT-1 T cell numbers in the inguinal lymph nodes 15-days post rAAV treatment. Recipient C57BL/6 mice received an initial adoptive transfer ratio of 9,000 tdTomato^{+} and 1,000 CD45.1^{+} gBT-1 T cells. Data are representative of two independent experiments with n=4 mice. (**g**) Quantification of the ratio of total tdTomato^{+}/CD45.1^{+} gBT-1 T cell numbers in the sinusoidal (S), portal tract (PT) and peri-central vein (PCV) compartments of the liver 12-days post rAAV treatment. Recipient C57BL/6 mice received an initial adoptive transfer of 1,000 CD45.1^{+} and 9,000 tdTomato^{+} gBT-1 T cells. Data points represent a single region of interest of each liver compartment from n = 8 mice from 2 independent experiments. Scale bars top panel 100µm, bottom panel 50µm (a-b), 100µm (c-d). Images are representative of n = 8 or 9 mice from 2 independent experiments (a, e). Analyzed with a Kruskal-Wallis test with multiple comparisons (g). ns = not statistically significant. Source data are provided as a Source Data file.

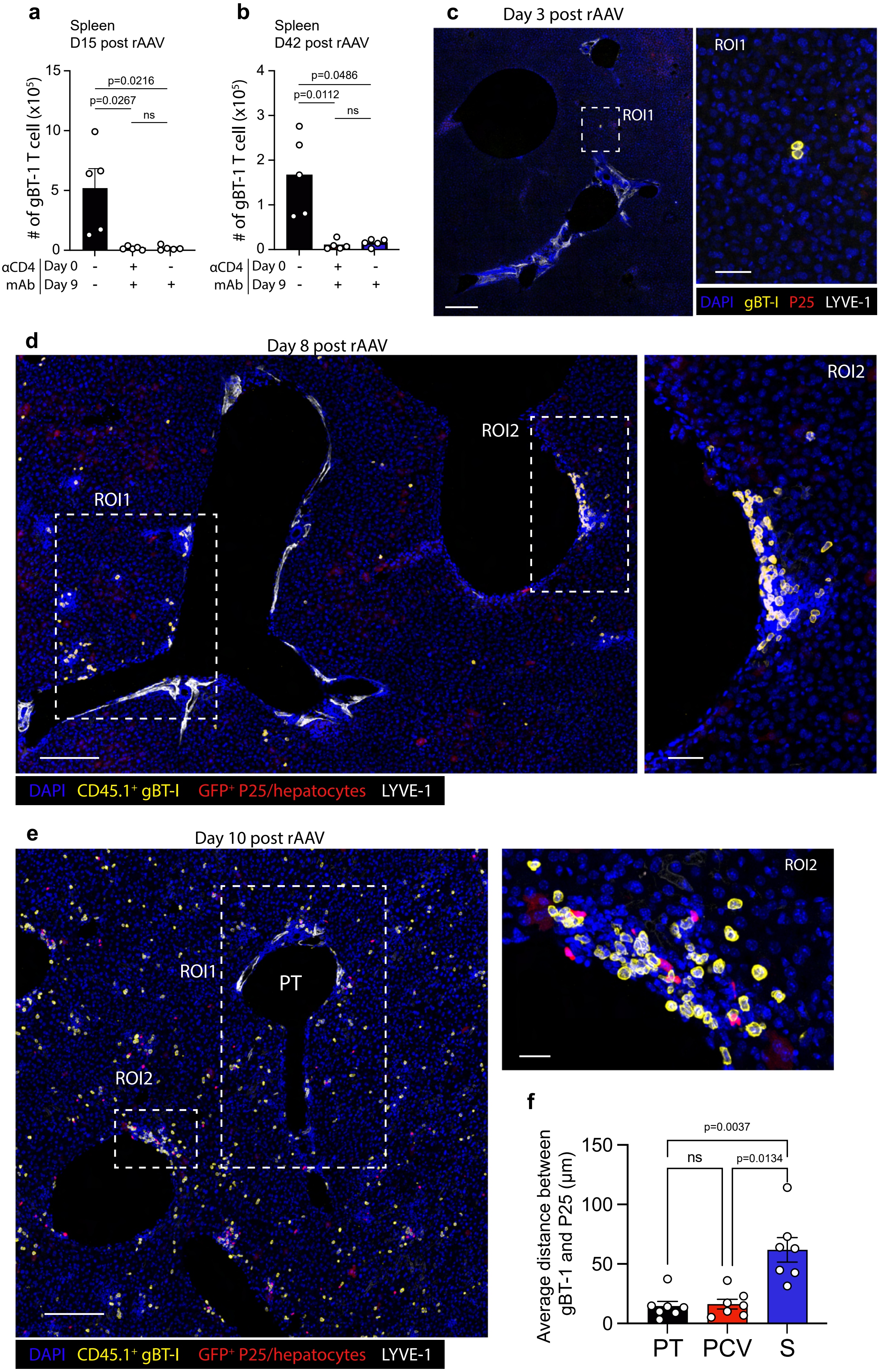

a
Spleen
D15 post rAAV
# of gBT-1 T cell (x10^5)
15
10
5
0
p=0.0216
p=0.0267
ns
αCD4
mAb
Day 0
Day 9
b
Spleen
D42 post rAAV
4
3
2
1
0
p=0.0486
p=0.0112
c
Day 3 post rAAV
ROI1
DAPI gBT-I P25 LYVE-1
d
Day 8 post rAAV
ROI1
ROI2
DAPI CD45.1+ gBT-I GFP+ P25/hepatocytes LYVE-1
e
Day 10 post rAAV
ROI1
ROI2
PT
DAPI CD45.1+ gBT-I GFP+ P25/hepatocytes LYVE-1
f
Average distance between gBT-1 and P25 (μm)
150
100
50
0
p=0.0037
p=0.0134
PT
PCV
S

Supplementary Figure 4. CD4 help signals are transferred in portal tracts and PCV regions of the liver. Related to Figure 4.

(**a-b**) Total number of splenic effector (a) and memory (b) gBT-1 T cells post rAAV treatment of recipient mice administered isotype control (shown as "-" in plots), or anti-CD4 depleting mAb starting at day 0 or at day 9. (**c-e**) Representative confocal images of gBT-1 and P25 T cells in a large region of the liver of rAAV treated mice at 3 days (c), 8 days (d) and 10 days (e) post rAAV treatment, showing that gBT-1 $CD8^+$ T cells were hardly detected in the liver before day 8 and that their migration preceded the migration of P25 $CD4^+$ T cells into the organ. $CD4^+$ T cells entered portal tracts and PCV regions, and interacted with cognate $CD8^+$ T cells after day 8. (d-e) ROI1 is shown with a higher magnification in Figure 4. PT = portal tract. Scale bars left panel 300µm, right panel 50µm (c), left panel 200µm, right panel 50µm (d), 200µm/25µm (e). (**f**) Quantification of the average distance (µm) between gBT-1 and P25 T cells in portal tracts (PT), peri-central vein (PCV) regions and the sinusoidal compartment (S) of the liver. n=4 or 5 mice over two independent experiments (a, b). Data points represent a single region of interest of each liver compartment from n = 7 mice from 2 independent experiments (f). Images are representative of n = 8 mice over two independent experiments (c, e). Error bars indicate ± SEM. Analysed with Kruskal-Wallis test with multiple comparisons (a, b, f). ns = not statistically significant. Source data are provided as a Source Data file.

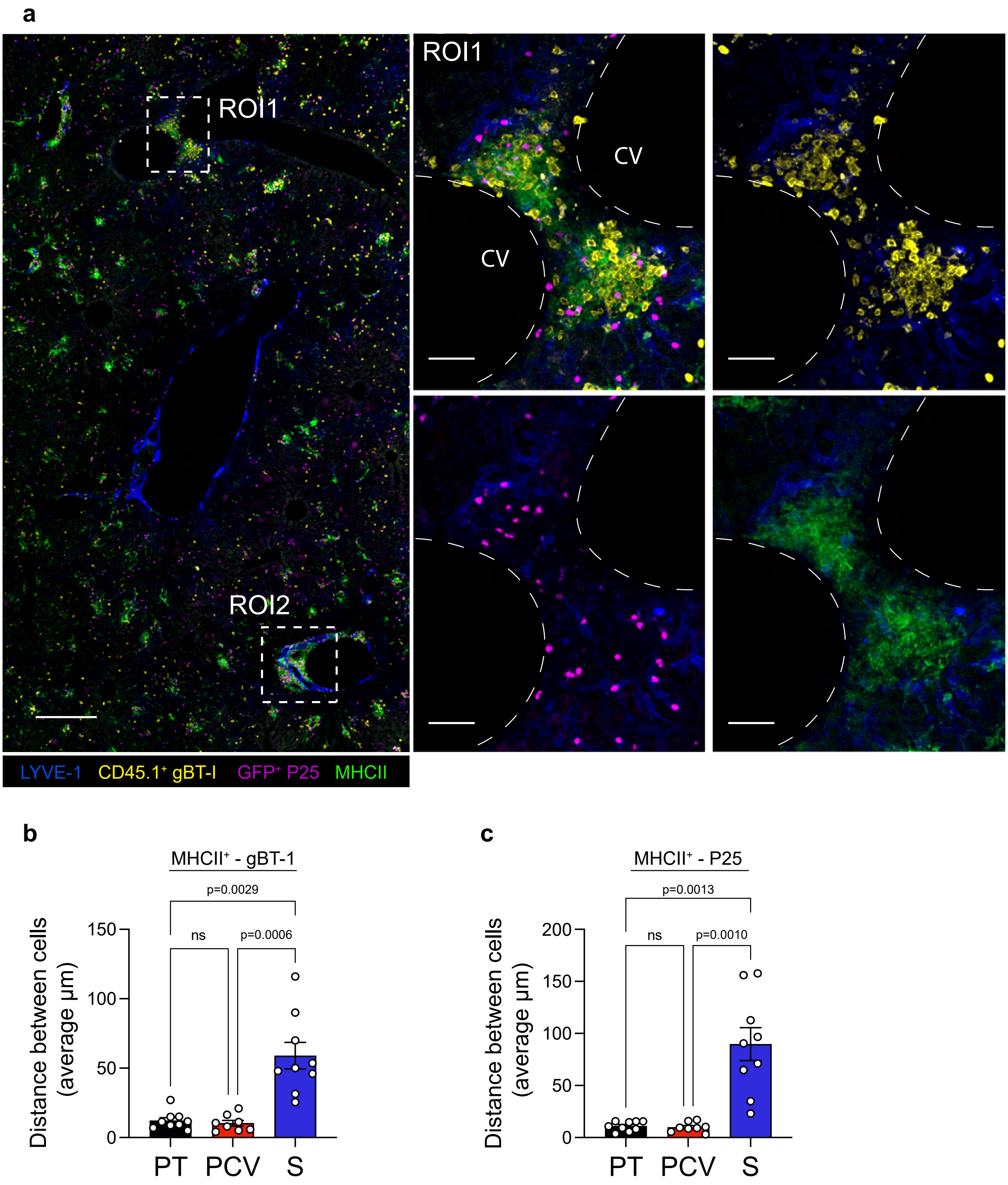
a
ROI1
ROI2
ROI1
CV
CV
LYVE-1 CD45.1⁺ gBT-I GFP⁺ P25 MHCII
b
MHCII⁺ - gBT-1
p=0.0029
ns
p=0.0006
Distance between cells (average µm)
150
100
50
0
PT PCV S
c
MHCII⁺ - P25
p=0.0013
ns
p=0.0010
Distance between cells (average µm)
200
150
100
50
0
PT PCV S

Supplementary Figure 5. Cognate $CD4^+$ and $CD8^+$ T cells cluster with $MHCII^{high}$ APCs in portal tracts and PCV regions. Related to Figure 5.

(**a**) Left panel: representative confocal image of a large region of the liver showing direct cell-cell interactions between gBT-1, P25 and $MHCII^{high}$ APCs in portal tracts and PCV regions 12-days post rAAV treatment. Right panels: magnified views of ROI1 showing individual and combined fluorescence stains for LYVE-1, MHCII, P25 (GFP^+) and gBT-1 ($CD45.1^+$) T cells. LYVE-1^{int} LSECs are observed surrounding the PCV area. ROI2 represents a portal tract region containing gBT-1, P25 and $MHCII^{high}$ APCs shown in high magnification in Figure 5D. CV = central vein. (**b-c**) Quantification of the average distance (μm) between gBT-1 T cells and $MHCII^{high}$ cells (b), and P25 T cells and $MHCII^{high}$ cells (b) in portal tracts (PT), peri-central vein (PCV) regions and the sinusoidal compartment (S) of the liver. Data points represent a single region of interest of each liver compartment from n=8 mice from 2 independent experiments (b, c). Scale bars left panel 300μm, right panels 50μm. Images are representative of two independent experiments with n=4 mice per group (a). Analysed with Kruskal-Wallis test with multiple comparisons (b, c), ns=not statistically significant. Source data are provided as a Source Data file.

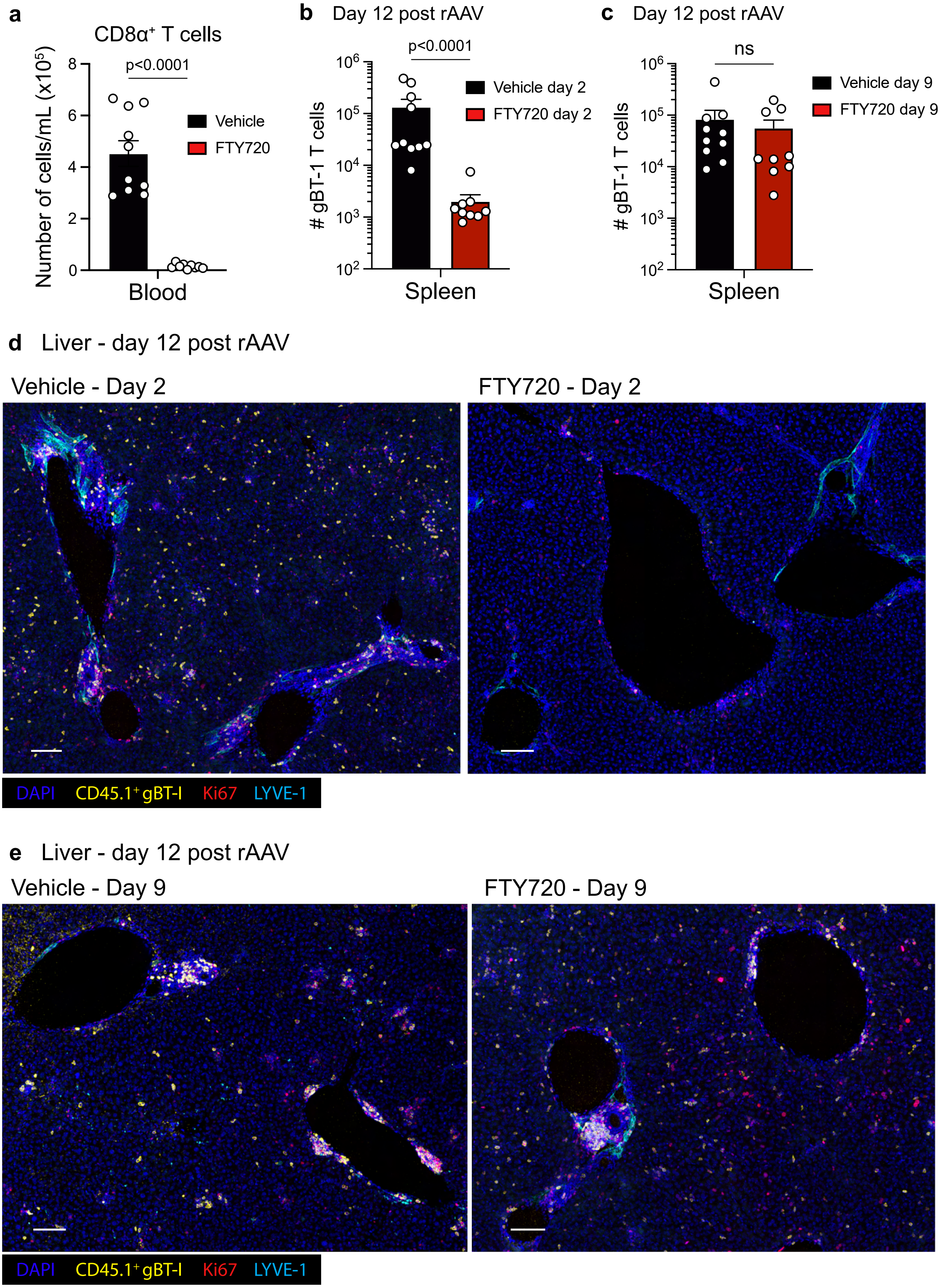
a
CD8α+ T cells
Number of cells/mL (x10^5)
p<0.0001
Vehicle
FTY720
Blood
b
Day 12 post rAAV
p<0.0001
Vehicle day 2
FTY720 day 2
gBT-1 T cells
Spleen
c
Day 12 post rAAV
ns
Vehicle day 9
FTY720 day 9
gBT-1 T cells
Spleen
d
Liver - day 12 post rAAV
Vehicle - Day 2
FTY720 - Day 2
DAPI
CD45.1+gBT-I
Ki67
LYVE-1
e
Liver - day 12 post rAAV
Vehicle - Day 9
FTY720 - Day 9
DAPI
CD45.1+gBT-I
Ki67
LYVE-1

Supplementary Figure 6. The expansion of intrahepatic CD8$^+$ T cells does require continued lymphoid tissue egress. Related to Figure 6.

(**a**) Quantification of the total number of endogenous CD8α$^+$ T cells in the blood of C57BL/6 mice treated with vehicle control (black) or FTY720 beginning 2-days post rAAV treatment. (**b**) Quantification of the total number of CD8$^+$ gBT-1 T cells in the spleens of recipient C57BL/6 mice treated with vehicle control (black) or FTY720 beginning 2-days post rAAV treatment. (**c**) Quantification of the total number of CD8$^+$ gBT-1 T cells in the spleens of recipient C57BL/6 mice treated with vehicle control (black) or FTY720 beginning 9-days post rAAV treatment. (**d-e**) Representative confocal IF images of Ki67$^+$ gBT-1 T cell clusters associated with portal tracts and PCV regions in the livers of mice treated with vehicle control (left panel) or FTY720 (right panel) beginning 2-days (d) or 9-days (e) post rAAV inoculation. Scale bar 100μm (d, e). n=9 or 10 mice from two independent experiments (a-c), images are representative of n=6 mice over two independent experiments. Error bars indicate ± SEM. Analysed with two-tailed unpaired Mann-Whitney U test, ns=not statistically significant. Source data are provided as a Source Data file.

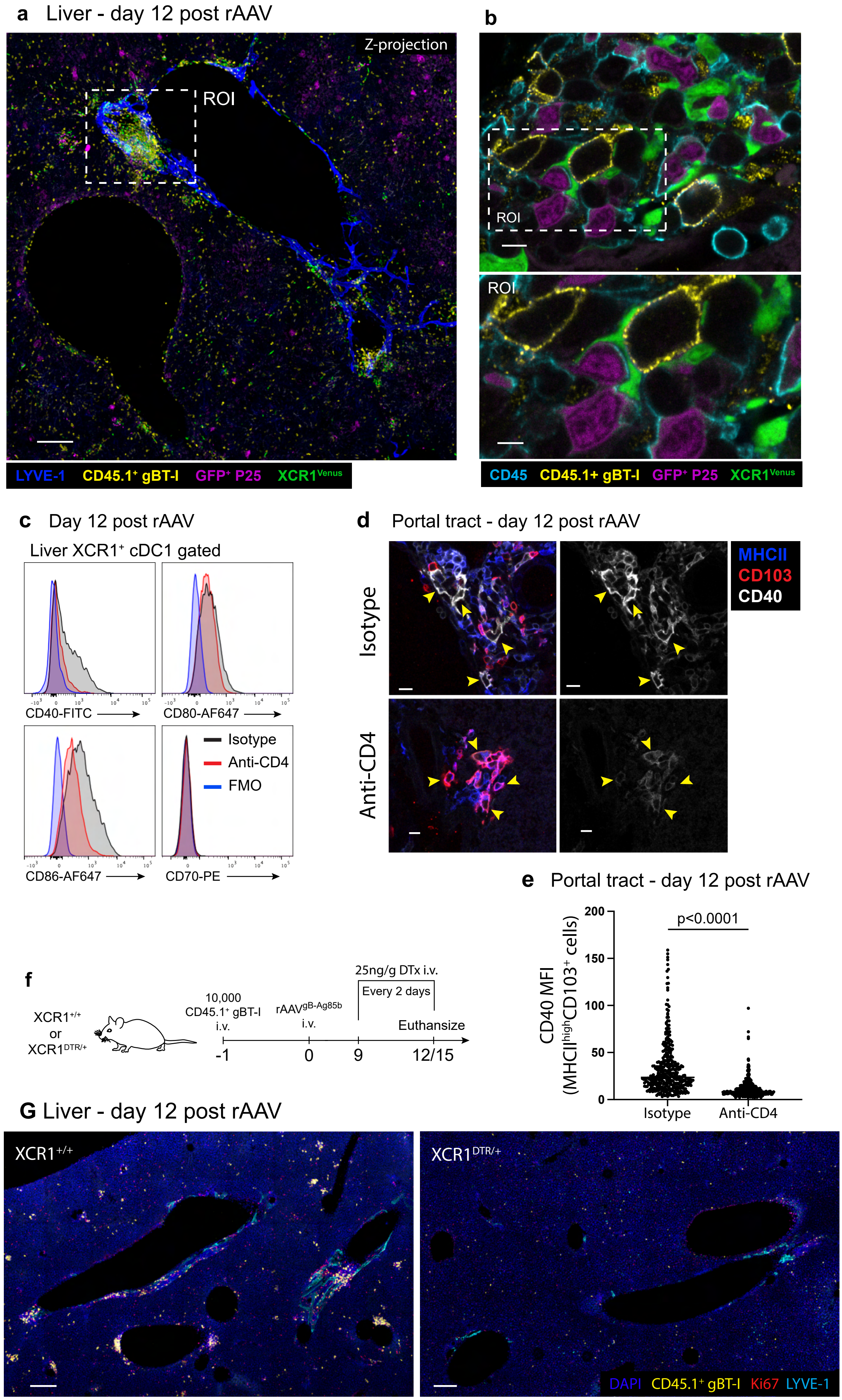

a Liver - day 12 post rAAV
Z-projection
ROI
LYVE-1 CD45.1+ gBT-I GFP+ P25 XCR1Venus
b
ROI
ROI
CD45 CD45.1+ gBT-I GFP+ P25 XCR1Venus
c Day 12 post rAAV
Liver XCR1+ cDC1 gated
CD40-FITC
CD80-AF647
CD86-AF647
CD70-PE
Isotype
Anti-CD4
FMO
d Portal tract - day 12 post rAAV
Isotype
Anti-CD4
MHCII
CD103
CD40
e Portal tract - day 12 post rAAV
CD40 MFI (MHCIIhighCD103+ cells)
p<0.0001
200
150
100
50
0
Isotype
Anti-CD4
f
XCR1+/+
or
XCR1DTR/+
10,000
CD45.1+ gBT-I
i.v.
rAAVgB-Ag85b
i.v.
25ng/g DTx i.v.
Every 2 days
Euthansize
-1
0
9
12/15
G Liver - day 12 post rAAV
XCR1+/+
XCR1DTR/+
DAPI CD45.1+ gBT-I Ki67 LYVE-1

Supplementary Figure 7. Cognate $CD4^+$ and $CD8^+$ T cells cluster with $XCR1^+$ cDC1 in portal tracts and PCV regions. Related to Figure 7.

(**a**) Representative confocal image of a large region of the liver showing gBT-1, P25 and $XCR1^+$ cDC1s in portal tracts and PCV regions 12-days post rAAV treatment of $XCR1^{venus/+}$ recipient mice. ROI1 highlights a portal tract (shown in Figure 7A) containing a large cluster of gBT-1 T cells (yellow), P25 T cells (magenta), and $XCR1^+$ cDC1s (green). (**b**) High magnification confocal image showing direct cell-cell interactions between gBT-1, P25 and $XCR1^+$ cDC1s in portal tracts 12-days post rAAV treatment of $XCR1^{venus/+}$ recipient mice. CD45 (cyan), gBT-1 T cells (yellow), P25 T cells (magenta), and $XCR1^+$ cDC1s (green). (**c**) Representative histograms showing the expression level of co-stimulatory molecules in $XCR1^+$ cDC1s isolated from the livers of CD4 depleted and isotype control treated recipient mice 12-days post $rAAV^{gB\text{-}Ag85b}$ treatment. (**d-e**) Representative confocal IF images (d) and quantification (e) of the mean fluorescence intensity (MFI) of CD40 on $MHCII^{high}CD103^+$ cDC1s in the portal tracts of recipient mice at 12-days post rAAV, following treatment with isotype control (top) or anti-CD4 mAb (bottom). Yellow arrowheads in (d) indicate $MHCII^{high}CD103^+$ cDC1s. Scale bar 10µm. Each data point represents a single cell from at least 2 portal tracts from n = 6 mice per group over 2 independent experiments (e). (**f - g**) Protocol (f) and representative confocal IF images showing $Ki67^+$ gBT-1 T cell clusters associated with portal tracts and PCV regions of the livers of $XCR1^{+/+}$ (left) and $XCR1^{DTR/+}$ (right) recipient mice at 12-days post rAAV, following treatment with diphtheria toxin (DTx) beginning 9-days post rAAV treatment (g). Scale bar 200µm (a and f), 4µm/2µm (b top/ROI bottom). Images are representative of n=6 or 8 mice over two independent experiments (a, b, d, g). Error bars indicate ± SEM. Analysed using a two-tailed unpaired Mann-Whitney test (e). Source data are provided as a Source Data file.

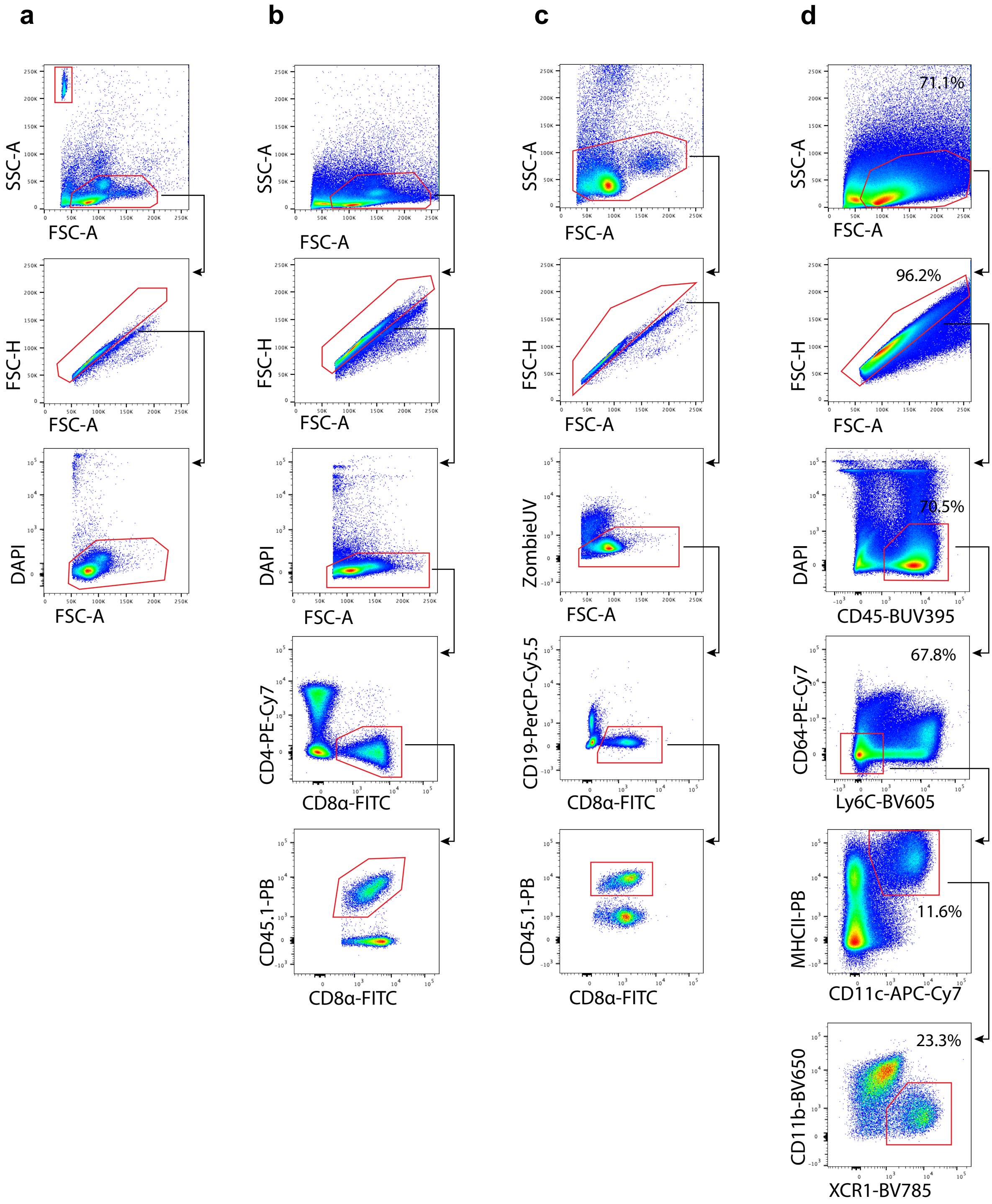
a
b
c
d
SSC-A
FSC-A
FSC-H
DAPI
CD4-PE-Cy7
CD8α-FITC
CD45.1-PB
ZombieUV
CD19-PerCP-Cy5.5
71.1%
96.2%
70.5%
CD45-BUV395
67.8%
CD64-PE-Cy7
Ly6C-BV605
MHCII-PB
11.6%
CD11c-APC-Cy7
23.3%
CD11b-BV650
XCR1-BV785

Supplementary Figure 8. Gating strategy used for flow cytometry analysis T cells and liver cDCs.

(**a**) Gating strategy to determine the total number of live cells in each sample. Lymphocytes and counting beads were gated using SSC-A and FSC-A profiles. Doublets were removed from the analysis using FSC-A vs FSC-H profiles and dead cells were excluded by eliminating cells positive for DAPI. (**b**) Gating strategy used to analyse T cell responses to rAAV in mice that had received an adoptive transfer of $CD45.1^{+}$ gBT-1 T cells before inoculation. Lymphocytes were gated using SSC-A and FSC-A profiles, doublets were removed from the analysis using FSC-A vs FSC-H, dead cells were excluded by eliminating cells positive for DAPI, $CD4^{-}$ cells were eliminated and donor gBT-1 T cells were selected by gating on $CD45.1^{+}$ cells. (**c**) Gating strategy used to analyse intracellular staining of T cells responding to rAAV in mice that had received an adoptive transfer of $CD45.1^{+}$ gBT-1 T cells before inoculation. Lymphocytes were gated using SSC-A and FSC-A profiles, doublets were removed from the analysis using FSC-A vs FSC-H, dead cells were excluded by eliminating cells positive for ZombieUV viability dye, B cells were eliminated by excluding $CD19^{+}$ cells, and donor gBT-1 T cells were selected by gating on $CD45.1^{+}$ cells. (**d**) Gating strategy used to analyse liver cDC responses to rAAV in mice. Leukocytes were gated using SSC-A and FSC-A profiles, doublets were removed from the analysis using FSC-A vs FSC-H, live cells of hematopoietic lineage were selected by excluding cells positive for DAPI and negative for CD45, $CD64^{-}Ly6C^{-}$ cells were selected to exclude liver macrophages and monocytes, $MHCII^{+}CD11c^{+}$ cells were gated to select conventional DCs and $XCR1^{+}$ cells were gated to select type-1 cDCs.

Supplementary Table 1: Antibodies used in this study.

Antigen/Fluorophore	Identifier	Source	Dilution
CD4-PE-Cy7	Clone RM4-4, AB_2563111	Biolegend	1/200
CD4-PE-Cy7	Clone RM4-5, AB_312729	Biolegend	1/200
CD8a-FITC	Clone 53-6.7, AB_312745	Biolegend	1/300
CD8a-BV650	Clone 53-6.7, AB_2738084	BD	1/300
CD11b-BV650	Clone M1/70, AB_2566568	Biolegend	1/200
CD11c-APC-Cy7	Clone N418, AB_830649	Biolegend	1/200
CD11c-BV711	Clone HL3, AB_2734778	BD	1/200
CD19-PerCP-Cy5.5	Clone 6D5, AB_2072925	Biolegend	1/300
CD25-APC	Clone PC-61, AB_312861	Biolegend	1/200
CD44-APC-Cy7	Clone IM7, AB_312963	Biolegend	1/200
CD45-BUV395	Clone 30-F11, AB_2651134	BD	1/300
CD45-BV421	Clone 30-F11, AB_10899570	Biolegend	1/200
CD45.1-PB	Clone A20, AB_492866	Biolegend	1/200
CD45.1-APC-Cy7	Clone A20, AB_313505	Biolegend	1/200
CD45.1-FITC	Clone A20, AB_313495	Biolegend	1/400
CD64-PE-Cy7	Clone X54-5/7.1, AB_2563904	Biolegend	1/200
CD69-APC	Clone H1.2F3, AB_492843	Biolegend	1/300
CD70-PE	Clone FR70, AB_313118	Biolegend	1/200
CD80-AF647	Clone 16-10A1, AB_492825	Biolegend	1/200
CD86-AF647	Clone GL-1, AB_493465	Biolegend	1/200
CD107a-AF647	Clone 1D4B, AB_571991	Biolegend	1/300
FITC purified	Rabbit polyclonal, AB_2533978	Invitrogen	1/400
Ki67-FITC	Clone SolA15, AB_11151330	Invitrogen	1/300
Ki67 purified	Clone SolA15, AB_10854564	Invitrogen	1/1000
LYVE-1 purified	Clone ALY7, AB_1633414	Invitrogen	1/1000
LYVE-1 eflour-570	Clone ALY7, AB_2573596	Invitrogen	1/400
GFP purified	Rabbit polyclonal, AB_2533978	Invitrogen	1/1000
GFP-AF488	Rabbit polyclonal, AB_2533978	Invitrogen	1/1500
MHCII (I-A/I-E)-PB	Clone M5/114.15.2, AB_493527	Biolegend	1/300
IFN-γ-PE	Clone XMG1.2, AB_395376	BD	1/300
Ly6C-BV605	Clone HK1.4, AB_2562353	Biolegend	1/200
XCR1-AF647	Clone ZET, AB_2564369	Biolegend	1/200
XCR1-BV785	Clone ZET, AB_2783119	Biolegend	1/200
Rat IgG-AF647	Goat polyclonal, AB_141778	Invitrogen	
Rabbit IgG-AF594	Donkey polyclonal, ab150076	abcam	
Rabbit IgG-AF488	Goat polyclonal, AB_143165	Invitrogen	
