# Supplementary material for: A hepatic network of dendritic cells mediates CD4 T cell help outside lymphoid organs
Source: Nat Commun. 2024 Feb 10;15:1261. doi: 10.1038/s41467-024-45612-5 (PMC10858872; doi:10.1038/s41467-024-45612-5)
Supplement: Supplementary file 3 — Reporting Summary [file 41467_2024_45612_MOESM3_ESM.pdf]

nature portfolio

Corresponding author(s): *Dr Patrick Bertolino and Dr David Bowen*

Last updated by author(s): *Jan 4, 2024*

Reporting Summary

Nature Portfolio wishes to improve the reproducibility of the work that we publish. This form provides structure for consistency and transparency in reporting. For further information on Nature Portfolio policies, see our Editorial Policies and the Editorial Policy Checklist.

Statistics

For all statistical analyses, confirm that the following items are present in the figure legend, table legend, main text, or Methods section.

n/a	Confirmed	
☐	☒	The exact sample size (n) for each experimental group/condition, given as a discrete number and unit of measurement
☐	☒	A statement on whether measurements were taken from distinct samples or whether the same sample was measured repeatedly
☐	☒	The statistical test(s) used AND whether they are one- or two-sided<br>*Only common tests should be described solely by name; describe more complex techniques in the Methods section.*
☒	☐	A description of all covariates tested
☐	☒	A description of any assumptions or corrections, such as tests of normality and adjustment for multiple comparisons
☐	☒	A full description of the statistical parameters including central tendency (e.g. means) or other basic estimates (e.g. regression coefficient) AND variation (e.g. standard deviation) or associated estimates of uncertainty (e.g. confidence intervals)
☐	☒	For null hypothesis testing, the test statistic (e.g. F, t, r) with confidence intervals, effect sizes, degrees of freedom and P value noted<br>*Give P values as exact values whenever suitable.*
☒	☐	For Bayesian analysis, information on the choice of priors and Markov chain Monte Carlo settings
☒	☐	For hierarchical and complex designs, identification of the appropriate level for tests and full reporting of outcomes
☒	☐	Estimates of effect sizes (e.g. Cohen's d, Pearson's r), indicating how they were calculated

Our web collection on statistics for biologists contains articles on many of the points above.

Software and code

Policy information about availability of computer code

Data collection	*For flow cytometry, samples were acquired using a LSR Fortessa with FACSDiva v9 software (Becton, Dickinson and Company, New Jersey, USA). Fluorescence microscopy data were collected using a Leica SP8 inverted confocal microscope with Leica Application Suite X software (Leica Microsystems, Wetzlar, Germany) .*
Data analysis	*Flow cytometry data was analyzed using Flowjo v10 software for MacOS (Ashland, OR; Becton, Dickinson and Company). Confocal microscopy data was analyzed using Imaris v9.6 software by Bitplane (Oxford Instruments, Abingdon, United Kingdom) with XTension plugin "Spots and Surfaces Distance" via MATLAB version: 9.13.0 (The MathWorks Inc. 2022. Natick, Massachusetts: The MathWorks Inc.). Numerical data was exported to Microsoft Excel v16.73 (Microsoft Corporation, Washington, USA) for further analysis. Prism v10 software (Graphpad software Inc.) was used for plotting graphs and performing statistical analysis.*

For manuscripts utilizing custom algorithms or software that are central to the research but not yet described in published literature, software must be made available to editors and reviewers. We strongly encourage code deposition in a community repository (e.g. GitHub). See the Nature Portfolio guidelines for submitting code & software for further information.

Data

Policy information about availability of data

All manuscripts must include a data availability statement. This statement should provide the following information, where applicable:

- Accession codes, unique identifiers, or web links for publicly available datasets
- A description of any restrictions on data availability
- For clinical datasets or third party data, please ensure that the statement adheres to our policy

Source data are provided with this paper. The data that support the findings of this study are available within the article, supplementary files, source data files or from the corresponding authors on request.

Research involving human participants, their data, or biological material

Policy information about studies with human participants or human data. See also policy information about sex, gender (identity/presentation), and sexual orientation and race, ethnicity and racism.

Reporting on sex and gender	*Use the terms sex (biological attribute) and gender (shaped by social and cultural circumstances) carefully in order to avoid confusing both terms. Indicate if findings apply to only one sex or gender; describe whether sex and gender were considered in study design; whether sex and/or gender was determined based on self-reporting or assigned and methods used. Provide in the source data disaggregated sex and gender data, where this information has been collected, and if consent has been obtained for sharing of individual-level data; provide overall numbers in this Reporting Summary. Please state if this information has not been collected. Report sex- and gender-based analyses where performed, justify reasons for lack of sex- and gender-based analysis.*
Reporting on race, ethnicity, or other socially relevant groupings	*Please specify the socially constructed or socially relevant categorization variable(s) used in your manuscript and explain why they were used. Please note that such variables should not be used as proxies for other socially constructed/relevant variables (for example, race or ethnicity should not be used as a proxy for socioeconomic status). Provide clear definitions of the relevant terms used, how they were provided (by the participants/respondents, the researchers, or third parties), and the method(s) used to classify people into the different categories (e.g. self-report, census or administrative data, social media data, etc.) Please provide details about how you controlled for confounding variables in your analyses.*
Population characteristics	*Describe the covariate-relevant population characteristics of the human research participants (e.g. age, genotypic information, past and current diagnosis and treatment categories). If you filled out the behavioural & social sciences study design questions and have nothing to add here, write "See above."*
Recruitment	*Describe how participants were recruited. Outline any potential self-selection bias or other biases that may be present and how these are likely to impact results.*
Ethics oversight	*Identify the organization(s) that approved the study protocol.*

Note that full information on the approval of the study protocol must also be provided in the manuscript.

Field-specific reporting

Please select the one below that is the best fit for your research. If you are not sure, read the appropriate sections before making your selection.

☒ Life sciences ☐ Behavioural & social sciences ☐ Ecological, evolutionary & environmental sciences

For a reference copy of the document with all sections, see nature.com/documents/nr-reporting-summary-flat.pdf

Life sciences study design

All studies must disclose on these points even when the disclosure is negative.

Sample size	*Sample sizes were chosen based on our prior experience and what is accepted in the field for in vivo experiments (References 21, 22 and 27). No statistical methods were used to calculate sample size. A minimum of three biological replicates were used for each experiment.*
Data exclusions	*No data was excluded unless explain by technical error.*
Replication	*Each experiment was reproduced at least two times to confirm reproducibility.*
Randomization	*Mice were randomly assigned to each group but were sex and age matched.*
Blinding	*Investigators were not blinded. Experiments were overseen by a lead researcher with full responsibility for animal manipulations. Data collection and analysis were therefore not performed blind to the conditions of the experiments.*

Behavioural & social sciences study design

All studies must disclose on these points even when the disclosure is negative.

Study description	
Research sample	*State the research sample (e.g. Harvard university undergraduates, villagers in rural India) and provide relevant demographic information (e.g. age, sex) and indicate whether the sample is representative. Provide a rationale for the study sample chosen. For studies involving existing datasets, please describe the dataset and source.*
Sampling strategy	*Describe the sampling procedure (e.g. random, snowball, stratified, convenience). Describe the statistical methods that were used to predetermine sample size OR if no sample-size calculation was performed, describe how sample sizes were chosen and provide a rationale for why these sample sizes are sufficient. For qualitative data, please indicate whether data saturation was considered, and what criteria were used to decide that no further sampling was needed.*
Data collection	*Provide details about the data collection procedure, including the instruments or devices used to record the data (e.g. pen and paper, computer, eye tracker, video or audio equipment) whether anyone was present besides the participant(s) and the researcher, and whether the researcher was blind to experimental condition and/or the study hypothesis during data collection.*
Timing	*Indicate the start and stop dates of data collection. If there is a gap between collection periods, state the dates for each sample cohort.*
Data exclusions	*If no data were excluded from the analyses, state so OR if data were excluded, provide the exact number of exclusions and the rationale behind them, indicating whether exclusion criteria were pre-established.*
Non-participation	*State how many participants dropped out/declined participation and the reason(s) given OR provide response rate OR state that no participants dropped out/declined participation.*
Randomization	*If participants were not allocated into experimental groups, state so OR describe how participants were allocated to groups, and if allocation was not random, describe how covariates were controlled.*

Ecological, evolutionary & environmental sciences study design

All studies must disclose on these points even when the disclosure is negative.

Study description	*Briefly describe the study. For quantitative data include treatment factors and interactions, design structure (e.g. factorial, nested, hierarchical), nature and number of experimental units and replicates.*
Research sample	*Describe the research sample (e.g. a group of tagged Passer domesticus, all Stenocereus thurberi within Organ Pipe Cactus National Monument), and provide a rationale for the sample choice. When relevant, describe the organism taxa, source, sex, age range and any manipulations. State what population the sample is meant to represent when applicable. For studies involving existing datasets, describe the data and its source.*
Sampling strategy	*Note the sampling procedure. Describe the statistical methods that were used to predetermine sample size OR if no sample-size calculation was performed, describe how sample sizes were chosen and provide a rationale for why these sample sizes are sufficient.*
Data collection	*Describe the data collection procedure, including who recorded the data and how.*
Timing and spatial scale	*Indicate the start and stop dates of data collection, noting the frequency and periodicity of sampling and providing a rationale for these choices. If there is a gap between collection periods, state the dates for each sample cohort. Specify the spatial scale from which the data are taken*
Data exclusions	*If no data were excluded from the analyses, state so OR if data were excluded, describe the exclusions and the rationale behind them, indicating whether exclusion criteria were pre-established.*
Reproducibility	*Describe the measures taken to verify the reproducibility of experimental findings. For each experiment, note whether any attempts to repeat the experiment failed OR state that all attempts to repeat the experiment were successful.*
Randomization	*Describe how samples/organisms/participants were allocated into groups. If allocation was not random, describe how covariates were controlled. If this is not relevant to your study, explain why.*
Blinding	*Describe the extent of blinding used during data acquisition and analysis. If blinding was not possible, describe why OR explain why blinding was not relevant to your study.*

Did the study involve field work? ☐ Yes ☒ No

Field work, collection and transport

Field conditions	*Describe the study conditions for field work, providing relevant parameters (e.g. temperature, rainfall).*
Location	*State the location of the sampling or experiment, providing relevant parameters (e.g. latitude and longitude, elevation, water depth).*
Access & import/export	*Describe the efforts you have made to access habitats and to collect and import/export your samples in a responsible manner and in compliance with local, national and international laws, noting any permits that were obtained (give the name of the issuing authority, the date of issue, and any identifying information).*
Disturbance	*Describe any disturbance caused by the study and how it was minimized.*

Reporting for specific materials, systems and methods

We require information from authors about some types of materials, experimental systems and methods used in many studies. Here, indicate whether each material, system or method listed is relevant to your study. If you are not sure if a list item applies to your research, read the appropriate section before selecting a response.

Materials & experimental systems

n/a	Involved in the study	
☐	☒	Antibodies
☒	☐	Eukaryotic cell lines
☒	☐	Palaeontology and archaeology
☐	☒	Animals and other organisms
☒	☐	Clinical data
☒	☐	Dual use research of concern
☒	☐	Plants

Methods

n/a	Involved in the study	
☒	☐	ChIP-seq
☐	☒	Flow cytometry
☒	☐	MRI-based neuroimaging

Antibodies

Antibodies used

Antigen/Fluorophore Identifier Source Dilution
CD4-PE-Cy7 Clone RM4-4, AB_2563111 Biolegend 1/200
CD4-PE-Cy7 Clone RM4-5, AB_312729 Biolegend 1/200
CD8a-FITC Clone 53-6.7, AB_312745 Biolegend 1/300
CD8a-BV650 Clone 53-6.7, AB_2738084 BD 1/300
CD11b-BV650 Clone M1/70, AB_2566568 Biolegend 1/200
CD11c-APC-Cy7 Clone N418, AB_830649 Biolegend 1/200
CD11c-BV711 Clone HL3, AB_2734778 BD 1/200
CD19-PerCP-Cy5.5 Clone 6D5, AB_2072925 Biolegend 1/300
CD25-APC Clone PC-61, AB_312861 Biolegend 1/200
CD44-APC-Cy7 Clone IM7, AB_312963 Biolegend 1/200
CD45-BUV395 Clone 30-F11, AB_2651134 BD 1/300
CD45-BV421 Clone 30-F11, AB_10899570 Biolegend 1/200
CD45.1-PB Clone A20, AB_492866 Biolegend 1/200
CD45.1-APC-Cy7 Clone A20, AB_313505 Biolegend 1/200
CD45.1-FITC Clone A20, AB_313495 Biolegend 1/400
CD64-PE-Cy7 Clone X54-5/7.1, AB_2563904 Biolegend 1/200
CD69-APC Clone H1.2F3, AB_492843 Biolegend 1/300
CD70-PE Clone FR70, AB_313118 Biolegend 1/200
CD80-AF647 Clone 16-10A1, AB_492825 Biolegend 1/200
CD86-AF647 Clone GL-1, AB_493465 Biolegend 1/200
CD107a-AF647 Clone 1D4B, AB_571991 Biolegend 1/300
FITC purified Rabbit polyclonal, AB_2533978 Invitrogen 1/400
Ki67-FITC Clone SolA15, AB_11151330 Invitrogen 1/300
Ki67 purified Clone SolA15, AB_10854564 Invitrogen 1/1000
LYVE-1 purified Clone ALY7, AB_1633414 Invitrogen 1/1000
LYVE-1 eflour-570 Clone ALY7, AB_2573596 Invitrogen 1/400
GFP purified Rabbit polyclonal, AB_2533978 Invitrogen 1/1000
GFP-AF488 Rabbit polyclonal, AB_2533978 Invitrogen 1/1500
MHCII (I-A/I-E)-PB Clone M5/114.15.2, AB_493527 Biolegend 1/300
IFN-γ-PE Clone XMG1.2, AB_395376 BD 1/300
Ly6C-BV605 Clone HK1.4, AB_2562353 Biolegend 1/200
XCR1-AF647 Clone ZET, AB_2564369 Biolegend 1/200
XCR1-BV785 Clone ZET, AB_2783119 Biolegend 1/200
Rat IgG-AF647 Goat polyclonal, AB_141778 Invitrogen

Rabbit IgG-AF594 Donkey polyclonal, ab150076 abcam
Rabbit IgG-AF488 Goat polyclonal, AB_143165 Invitrogen

Validation

All antibodies are from commercial sources and have been validated by the vendors. Validation data are available on the manufacturer's websites (summarized below). Antibodies have been titrated by the authors to find the optimal dilution for staining.
Invitrogen and eBioscience antibodies for flow cytometry and immunofluorescnce histology from Thermofisher Scientific undergo target validation with a two-part system that includes target specificity verification and functional application validation. Target specificity verification can involve one or more method, examples include but are not limited to target knockout using CRISPRCas9
and neutralization using blocking antibodies. Functional application validation assesses performance in the given application (i.e. flow cytometry). https://www.thermofisher.com/nz/en/home/life-science/antibodies/invitrogen-antibody-validation.html.
Antibodies are tested with mouse bone marrow cells, splenocytes and/or thymocytes.
Antibodies used for flow cytometry from Biolegend were validated by the supplier for specificity using 1-3 cell lines that are target-positive or -negative using single- or multi-colour flow cytometry analysis. Each new lot of antibody undergoes QC analysis by confirming brightness by MFI compared to the in-date reference lot. https://www.biolegend.com/en-us/quality/quality-control.
BD Biosciences antibodies (including BD OptiBuildTM antibodies) undergo multiple methodologies including flow cytometry, immunofluorescence, immunohistochemistry or western blot on a combination of primary cells, cell lines or transfectant models to confirm specificity. https://www.bdbiosciences.com/en-us/products/reagents/flow-cytometry-reagents/research-reagents/qualityand-reproducibility.
Antibodies used for immunofluorescence histology from Abcam were validated by the supplier. Initially, scientists will review the available literature to determine the best cell lines and tissues to use for validation. Antibodies are then tested by staining multiple normal human tissue microarrays (TMAs), multi-tumor human TMAs, and rat or mouse TMAs during antibody development.

Eukaryotic cell lines

Policy information about cell lines and Sex and Gender in Research

Cell line source(s)

State the source of each cell line used and the sex of all primary cell lines and cells derived from human participants or vertebrate models.

Authentication

Describe the authentication procedures for each cell line used OR declare that none of the cell lines used were authenticated.

Mycoplasma contamination

Confirm that all cell lines tested negative for mycoplasma contamination OR describe the results of the testing for mycoplasma contamination OR declare that the cell lines were not tested for mycoplasma contamination.

Commonly misidentified lines
(See ICLAC register)

Name any commonly misidentified cell lines used in the study and provide a rationale for their use.

Palaeontology and Archaeology

Specimen provenance

Provide provenance information for specimens and describe permits that were obtained for the work (including the name of the issuing authority, the date of issue, and any identifying information). Permits should encompass collection and, where applicable, export.

Specimen deposition

Indicate where the specimens have been deposited to permit free access by other researchers.

Dating methods

If new dates are provided, describe how they were obtained (e.g. collection, storage, sample pretreatment and measurement), where they were obtained (i.e. lab name), the calibration program and the protocol for quality assurance OR state that no new dates are provided.

☐ Tick this box to confirm that the raw and calibrated dates are available in the paper or in Supplementary Information.

Ethics oversight

Identify the organization(s) that approved or provided guidance on the study protocol, OR state that no ethical approval or guidance was required and explain why not.

Note that full information on the approval of the study protocol must also be provided in the manuscript.

Animals and other research organisms

Policy information about studies involving animals; ARRIVE guidelines recommended for reporting animal research, and Sex and Gender in Research

Laboratory animals

Mouse strains used were C57BL/6J, gBT-1, P25, Rag1-/-, CD45.1, GFP, mT/mG, XCR1-Venus and XCR1-DTRvenus. Mice were housed under 12 hour light/ 12 hour dark cycle, at temperatures of 22+/-2 degrees Celcius with 50% +/- 10% humidity. Experiments were performed on mice aged between 6 and 12 weeks of age.

Wild animals

No wild animals were used in the study.

Reporting on sex

Reporting on sex differences has not been collected. We used both female and male mice in this study. In all experiments, mice were

Reporting on sex	*sex matched between groups. We did not observe any significant differences between the outcome of experiments using females compared to males.*
Field-collected samples	*No field collected samples were used in the study.*
Ethics oversight	*Animal ethics protocols were approved by the Sydney Local Health District Animal Welfare Ethics Committee (Royal Prince Alfred Hospital, Sydney, Australia). (Protocols 2020-005, 2021-013, 2016-006, 2016-041).*

Note that full information on the approval of the study protocol must also be provided in the manuscript.

Clinical data

Policy information about clinical studies

All manuscripts should comply with the ICMJE guidelines for publication of clinical research and a completed CONSORT checklist must be included with all submissions.

Clinical trial registration	*Provide the trial registration number from ClinicalTrials.gov or an equivalent agency.*
Study protocol	*Note where the full trial protocol can be accessed OR if not available, explain why.*
Data collection	*Describe the settings and locales of data collection, noting the time periods of recruitment and data collection.*
Outcomes	*Describe how you pre-defined primary and secondary outcome measures and how you assessed these measures.*

Dual use research of concern

Policy information about dual use research of concern

Hazards

Could the accidental, deliberate or reckless misuse of agents or technologies generated in the work, or the application of information presented in the manuscript, pose a threat to:

No	Yes	
☒	☐	Public health
☒	☐	National security
☒	☐	Crops and/or livestock
☒	☐	Ecosystems
☒	☐	Any other significant area

Experiments of concern

Does the work involve any of these experiments of concern:

No	Yes	
☒	☐	Demonstrate how to render a vaccine ineffective
☒	☐	Confer resistance to therapeutically useful antibiotics or antiviral agents
☒	☐	Enhance the virulence of a pathogen or render a nonpathogen virulent
☒	☐	Increase transmissibility of a pathogen
☒	☐	Alter the host range of a pathogen
☒	☐	Enable evasion of diagnostic/detection modalities
☒	☐	Enable the weaponization of a biological agent or toxin
☒	☐	Any other potentially harmful combination of experiments and agents

Plants

Seed stocks | *Report on the source of all seed stocks or other plant material used. If applicable, state the seed stock centre and catalogue number. If plant specimens were collected from the field, describe the collection location, date and sampling procedures.*

Novel plant genotypes | *Describe the methods by which all novel plant genotypes were produced. This includes those generated by transgenic approaches, gene editing, chemical/radiation-based mutagenesis and hybridization. For transgenic lines, describe the transformation method, the number of independent lines analyzed and the generation upon which experiments were performed. For gene-edited lines, describe the editor used, the endogenous sequence targeted for editing, the targeting guide RNA sequence (if applicable) and how the editor was applied.*

Authentication | *Describe any authentication procedures for each seed stock used or novel genotype generated. Describe any experiments used to assess the effect of a mutation and, where applicable, how potential secondary effects (e.g. second site T-DNA insertions, mosiacism, off-target gene editing) were examined.*

ChIP-seq

Data deposition

☐ Confirm that both raw and final processed data have been deposited in a public database such as GEO.

☐ Confirm that you have deposited or provided access to graph files (e.g. BED files) for the called peaks.

Data access links
May remain private before publication. | *For "Initial submission" or "Revised version" documents, provide reviewer access links. For your "Final submission" document, provide a link to the deposited data.*

Files in database submission | *Provide a list of all files available in the database submission.*

Genome browser session
(e.g. UCSC) | *Provide a link to an anonymized genome browser session for "Initial submission" and "Revised version" documents only, to enable peer review. Write "no longer applicable" for "Final submission" documents.*

Methodology

Replicates | *Describe the experimental replicates, specifying number, type and replicate agreement.*

Sequencing depth | *Describe the sequencing depth for each experiment, providing the total number of reads, uniquely mapped reads, length of reads and whether they were paired- or single-end.*

Antibodies | *Describe the antibodies used for the ChIP-seq experiments; as applicable, provide supplier name, catalog number, clone name, and lot number.*

Peak calling parameters | *Specify the command line program and parameters used for read mapping and peak calling, including the ChIP, control and index files used.*

Data quality | *Describe the methods used to ensure data quality in full detail, including how many peaks are at FDR 5% and above 5-fold enrichment.*

Software | *Describe the software used to collect and analyze the ChIP-seq data. For custom code that has been deposited into a community repository, provide accession details.*

Flow Cytometry

Plots

Confirm that:

☒ The axis labels state the marker and fluorochrome used (e.g. CD4-FITC).

☒ The axis scales are clearly visible. Include numbers along axes only for bottom left plot of group (a 'group' is an analysis of identical markers).

☒ All plots are contour plots with outliers or pseudocolor plots.

☒ A numerical value for number of cells or percentage (with statistics) is provided.

Methodology

Sample preparation | *To isolate hepatocytes, the portal vein was transection to allow perfusate outflow and the liver was perfused in situ retrogradely via the IVC using a 4.5mL/min flow by sequentially injecting the following solutions prewarmed at 37OC: 25mL of HBSS, followed by 25mL HBSS with 0.5 mM EDTA, 25mL HBSS, and finally 25 mL HBSS containing 1 mg/mL collagenase type IV (Sigma-Aldrich, St. Louis, MI, USA) and 5 mM CaCl2.21 After the final perfusion, gallbladders were excised, livers were removed en bloc and hepatocytes were gently teased from the liver into RPMI + 5% FCS where they were kept on ice for further analysis.*

To isolate liver lymphocytes, livers were retrogradely perfused in situ with 5ml of PBS via the IVC, following transection of the portal vein to allow outflow of perfusate.21 Gallbladders were excised, and livers were then removed en bloc. Livers were gently pressed through an 80-gauge stainless steel mesh sieve in RPMI + 2% FCS, then centrifuged at 500g for 5 minutes at 4OC. The pellet was resuspended in a solution of 36% isotonic Percoll (GE Life Sciences, Parramatta, NSW, Australia) in PBS solution and centrifuged at 800g for 12 minutes at 4OC. The cell pellet was washed with RPMI + 2% FCS followed by red cell removal buffer (150 mM ammonium chloride, 0.1 mM EDTA disodium, 12 mM sodium hydrogen carbonate in triple distilled water), followed by a further wash in RPMI + 2% FCS. Lymphocytes were isolated from lymph nodes and spleens by pressing the tissue through an 80-gauge stainless steel sieve and washing in RPMI + 2% FCS.
To isolate GI Tract lymphocytes, the small intestine, large intestine, and Peyer's patch tissues were treated sequentially with PBS containing 1 mM DTT at room temperature for 10 min, twice with 5 mM EDTA at 37 °C for 10 min to remove epithelial cells, and then minced and dissociated in digestion buffer (RPMI containing collagenase (1 mg/ml collagenase D; Roche), DNase I (200 μg/mL; Sigma) with constant stirring at 37 °C 55 min. 60 Leukocytes were collected at the interface of a 40%–80% Percoll gradient (GE Healthcare). For blood lymphocytes, blood was collected by cardiac puncture into tubes containing Alsever's solution (Sigma-Aldrich, St. Louis, MI, USA). Peripheral blood mononuclear cells (PBMC) were prepared by lysing red blood cells with two washes of red cell removal buffer followed by washing with RPMI + 2% FCS. Single cell suspensions were stored in RPMI + 2% FCS on ice for further analysis.

For liver dendritic cells, Livers were retrogradely perfused in situ with 5ml of 37OC HBSS containing Ca and Mg, as well as 1mg/mL collagenase type IV (Sigma-Aldrich, St. Louis, MI, USA) via the IVC, following transection of the portal vein to allow outflow of perfusate. Gallbladders were excised, and livers were then removed, chopped finely and incubated for 30 minutes in HBSS with Ca and Mg, containing 1mg/mL collagenase type IV and 1ug/mL DNase type I (Roche, Basel, Switzerland) at 37OC. Livers were gently pressed through an 80-gauge stainless steel mesh sieve in RPMI + 2% FCS, then centrifuged at 440g for 5 minutes at 4OC. The pellet was resuspended in a solution of 33% isotonic Percoll (GE Life Sciences, Parramatta, NSW, Australia) in PBS solution and centrifuged at 700g for 12 minutes at room temperature with no brake. The cell pellet was washed with RPMI + 2% FCS followed by red cell removal buffer, followed by a further wash in RPMI + 2% FCS. Single cell suspensions were stored in RPMI + 2% FCS on ice for further analysis.

Instrument

BD LSR Fortessa (Becton, Dickinson and Company, USA).

Software

BD FACSDiva v9 (Becton, Dickinson and Company, USA).

Cell population abundance

gBT-1.Ly5.1.Rag1-/- and P25.GFP.Rag1-/- cells used for adoptive transfer experiments were counted using a hemocytometer. Sort purity following FACS was determined by running post sort sample.

Gating strategy

For all analyses, cells were first gated on time, then FSC X SSC for lymphocytes, singlet discrimination gates (FSC-A x FSC-H) and live cells (DAPI, ZombieUV or Viability Dye 700 negative). IN figure 1, 2, 4, 5, 6, and 7, donor gBT-1.L75.1.Rag1-/- cells were then selected by gating on CD8a+CD45.1+ cells. In figure 1, donor P25.GFP.Rag1-/- cells were selected by gating on CD4 +GFP+ cells. For intracellular cytokine and transcription factor staining, donor gBT-1.Ly5.1.Rag1-/- cells were assessed after removing cells binding to CD19.
For liver dendritic cells, CD45+ cells were selected before removal of Ly6C+ and CD64+ populations. Liver dendritic cells were selected by gating on CD11c+, MHCII+ cells. Type-1 conventional dendritic cells were selected by gating on XCR1+ cells.

☒ Tick this box to confirm that a figure exemplifying the gating strategy is provided in the Supplementary Information.

Magnetic resonance imaging

Experimental design

Design type

Indicate task or resting state; event-related or block design.

Design specifications

Specify the number of blocks, trials or experimental units per session and/or subject, and specify the length of each trial or block (if trials are blocked) and interval between trials.

Behavioral performance measures

State number and/or type of variables recorded (e.g. correct button press, response time) and what statistics were used to establish that the subjects were performing the task as expected (e.g. mean, range, and/or standard deviation across subjects).

Acquisition

Imaging type(s)

Specify: functional, structural, diffusion, perfusion.

Field strength

Specify in Tesla

Sequence & imaging parameters

Specify the pulse sequence type (gradient echo, spin echo, etc.), imaging type (EPI, spiral, etc.), field of view, matrix size, slice thickness, orientation and TE/TR/flip angle.

Area of acquisition

State whether a whole brain scan was used OR define the area of acquisition, describing how the region was determined.

Diffusion MRI ☐ Used ☐ Not used

Preprocessing

Preprocessing software | *Provide detail on software version and revision number and on specific parameters (model/functions, brain extraction, segmentation, smoothing kernel size, etc.).*

Normalization | *If data were normalized/standardized, describe the approach(es): specify linear or non-linear and define image types used for transformation OR indicate that data were not normalized and explain rationale for lack of normalization.*

Normalization template | *Describe the template used for normalization/transformation, specifying subject space or group standardized space (e.g. original Talairach, MNI305, ICBM152) OR indicate that the data were not normalized.*

Noise and artifact removal | *Describe your procedure(s) for artifact and structured noise removal, specifying motion parameters, tissue signals and physiological signals (heart rate, respiration).*

Volume censoring | *Define your software and/or method and criteria for volume censoring, and state the extent of such censoring.*

Statistical modeling & inference

Model type and settings | *Specify type (mass univariate, multivariate, RSA, predictive, etc.) and describe essential details of the model at the first and second levels (e.g. fixed, random or mixed effects; drift or auto-correlation).*

Effect(s) tested | *Define precise effect in terms of the task or stimulus conditions instead of psychological concepts and indicate whether ANOVA or factorial designs were used.*

Specify type of analysis: ☐ Whole brain ☐ ROI-based ☐ Both

Statistic type for inference | *Specify voxel-wise or cluster-wise and report all relevant parameters for cluster-wise methods.*

(See Eklund et al. 2016)

Correction | *Describe the type of correction and how it is obtained for multiple comparisons (e.g. FWE, FDR, permutation or Monte Carlo).*

Models & analysis

n/a	Involved in the study
☐	☐ Functional and/or effective connectivity
☐	☐ Graph analysis
☐	☐ Multivariate modeling or predictive analysis

Functional and/or effective connectivity | *Report the measures of dependence used and the model details (e.g. Pearson correlation, partial correlation, mutual information).*

Graph analysis | *Report the dependent variable and connectivity measure, specifying weighted graph or binarized graph, subject- or group-level, and the global and/or node summaries used (e.g. clustering coefficient, efficiency, etc.).*

Multivariate modeling and predictive analysis | *Specify independent variables, features extraction and dimension reduction, model, training and evaluation metrics.*
